# Evaluation of machine learning and deep learning algorithms for fire prediction in Southeast Asia

**DOI:** 10.1038/s41598-025-00628-9

**Published:** 2025-05-29

**Authors:** Aditya Eaturu, Krishna Prasad Vadrevu

**Affiliations:** 1 University of Alabama, Huntsville, USA; 2 NASA Marshall Space Flight Center, Huntsville, AL USA

**Keywords:** Environmental sciences, Environmental impact

## Abstract

Vegetation fires are most common in Southeast Asian (SEA) countries, causing biodiversity loss, habitat destruction, and air pollution. Accurately predicting fire occurrences in SEA remains challenging due to its complex spatiotemporal dynamics. Improved fire predictions enable timely interventions, helping to control and mitigate fires. In this study, we utilize Visible Infrared Imaging Radiometer Suite (VIIRS) satellite-derived fire data alongside six machine learning (ML) and deep learning (DL) models, Simple Persistence, Multi-Layer Perceptron (MLP), Convolutional Neural Network (CNN), Long Short-Term Memory (LSTM), CNN-Long Short-Term Memory (CNN-LSTM), and Convolutional Long Short-Term Memory (ConvLSTM) to determine the most effective fire prediction model. We evaluated model performance using Root Mean Square Error (RMSE), Mean Absolute Error (MAE), and R^2^ (coefficient of determination). Our results indicate that the CNN performs best in regions with strong spatial dependencies, such as Brunei, Indonesia, Malaysia, the Philippines, Timor-Leste, and Thailand. Conversely, the ConvLSTM excels in countries with complex spatiotemporal dynamics, like Laos, Myanmar, and Vietnam. The CNN-LSTM hybrid model also performed well in Cambodia, suggesting a need for a balanced approach in areas requiring both spatial and temporal feature extraction. Furthermore, simpler models, such as Simple Persistence and MLP, showed limitations in capturing dynamic patterns and temporal dependencies. Our findings highlight the importance of evaluating various ML and DL models before integrating them into any decision support systems (DSS) for fire management studies. By tailoring models to specific regional fire data, prediction accuracy and responsiveness can be enhanced, ultimately improving fire risk management in Southeast Asia and beyond.

## Introduction

Fires are a common natural occurrence across many landscapes^1^. However, fire regimes differ worldwide^2^ and are primarily influenced by variations in the type of fuels available, local weather, topography, and human factors^3^. Notably, tropical ecosystems, which constitute only 16% of the Earth’s land area, account for 78% of the global area affected by fire. Therefore, shifts in fire regimes within these tropical regions can have significant and uneven impacts on terrestrial and atmospheric systems. In South and Southeast Asian countries, human activities and natural causes, such as lightning, are drivers of fires^4^. For instance, fire is frequently employed as a method for land clearing through slash-and-burn techniques^5,6^. Additionally, burning crop residues is a prevalent practice for disposing of leftover materials after harvests and preparing land for new crops. Fires are also utilized for hunting, pest management, and facilitating livestock movement^7,8^. Regardless of their causes, fires can severely damage ecosystems and human communities. They can lead to complete or partial vegetation loss, altering plant composition, structure, and function, disrupting biogeochemical cycles, and resulting in nutrient depletion. Frequent burning in forested areas may lead to pyro denitrification^9^. Research indicates that landscapes subject to repeated fires tend to have soils with low nutrient levels, particularly reductions in nitrogen, which impact soil fertility and long-term ecosystem productivity. Another significant consequence of fires is the emission of greenhouse gases and aerosols, which can directly and indirectly influence climate^10,11^. The effects of biomass burning, particularly concerning the pollutants released, are contingent on various factors, including the type of vegetation involved, the quantity burned, fire frequency and intensity, and, crucially, the efficiency of the burning process^12^.

Ground-based methods for studying fires and assessing their environmental impacts have significant limitations due to spatial and temporal variability influenced by climate, vegetation, topography, and various land use and cover changes. Additionally, safety concerns can hinder ground-based assessments^13^. Monitoring fire occurrences through traditional surveys can be particularly challenging, as fires often occur in remote, hard-to-reach areas. In contrast, timely fire activity and behavior updates are crucial for effective management and mitigation strategies. In recent years, remote sensing and geospatial technologies have emerged as powerful tools for detecting, mapping, and monitoring fires^14^. These technologies provide essential data for fire management, thanks to their ability to capture multispectral, multi-temporal, synoptic, and repetitive coverage of large areas^15^. When combined with advanced spatial algorithms, remote sensing data can effectively track the spatial and temporal variations of fires and their impacts on both land and atmospheric conditions^16^. Furthermore, this technology is instrumental in assessing post-fire land cover changes^17^, which can inform global fire control strategies across different regions. Integrating remote sensing technologies enhances our understanding of fire dynamics and supports more responsive and adaptive management practices. By providing critical information on fire patterns, severity, and ecological effects, these tools enable stakeholders to make informed decisions to mitigate fire risks and manage the aftermath of wildfires effectively. This evolution in fire monitoring represents a significant advancement in addressing the challenges of wildfires in various landscapes worldwide.

Accurate fire prediction safeguards ecosystems, public health, and infrastructure. Traditional models often fall short in this area, particularly in regions with complex and dynamic fire patterns, as they struggle to account for the intricate, time-dependent behaviors of fire events. In contrast, machine learning and deep learning models excel at processing vast datasets and uncovering hidden patterns, positioning them as a promising alternative for fire prediction. Further, satellite data has advantages as it can provide real-time, high-resolution data on fire characteristics to monitor areas prone to fire and assess fire behavior more effectively. Integrating remote sensing data with machine learning and deep learning algorithms allows for a more comprehensive analysis, as these models can leverage the rich temporal and spatial information captured by satellites. This synergy can improve the accuracy of fire predictions and facilitate early warning systems, helping communities and decision-makers respond more effectively to emerging fire threats.

In Southeast Asia, integrating satellite data with machine learning and deep learning methods for fire prediction and modeling remains an underexplored area. While some studies have focused on fire mapping and monitoring, including their impacts, the application of advanced computational techniques alongside remote sensing data for understanding fire prediction is still relatively scarce. This gap is particularly concerning given the region’s unique ecological and climatic conditions, which render its ecosystems highly vulnerable to the effects of wildfires.

In this study, we present a comprehensive comparison of six different machine learning and deep learning models: Simple Persistence, Multi-Layer Perceptron (MLP), Convolutional Neural Network (CNN), Long Short-Term Memory (LSTM), CNN-LSTM, and ConvLSTM, for fire prediction in ten different Southeast Asian countries using consistent evaluation metrics and a uniform dataset which has not been previously attempted. Our goal is to determine which model provides the most accurate fire predictions. To conduct this analysis, we focused on Southeast Asian countries where vegetation fires are common but show distinct spatial and temporal trends. We analyzed satellite-derived fire data for model evaluation. Additionally, we validated the results statistically by comparing them with independent datasets and employed robust statistical metrics to verify model performance. Projections were also conducted for an additional five years. The findings from this research aim to identify the most effective fire prediction model using satellite data. By delineating the strengths and weaknesses of each model, this study contributes to advancing fire prediction techniques, ultimately enhancing our ability to mitigate the impacts of wildfires in various regions around the world.

## Study area

**Fig. 1:**
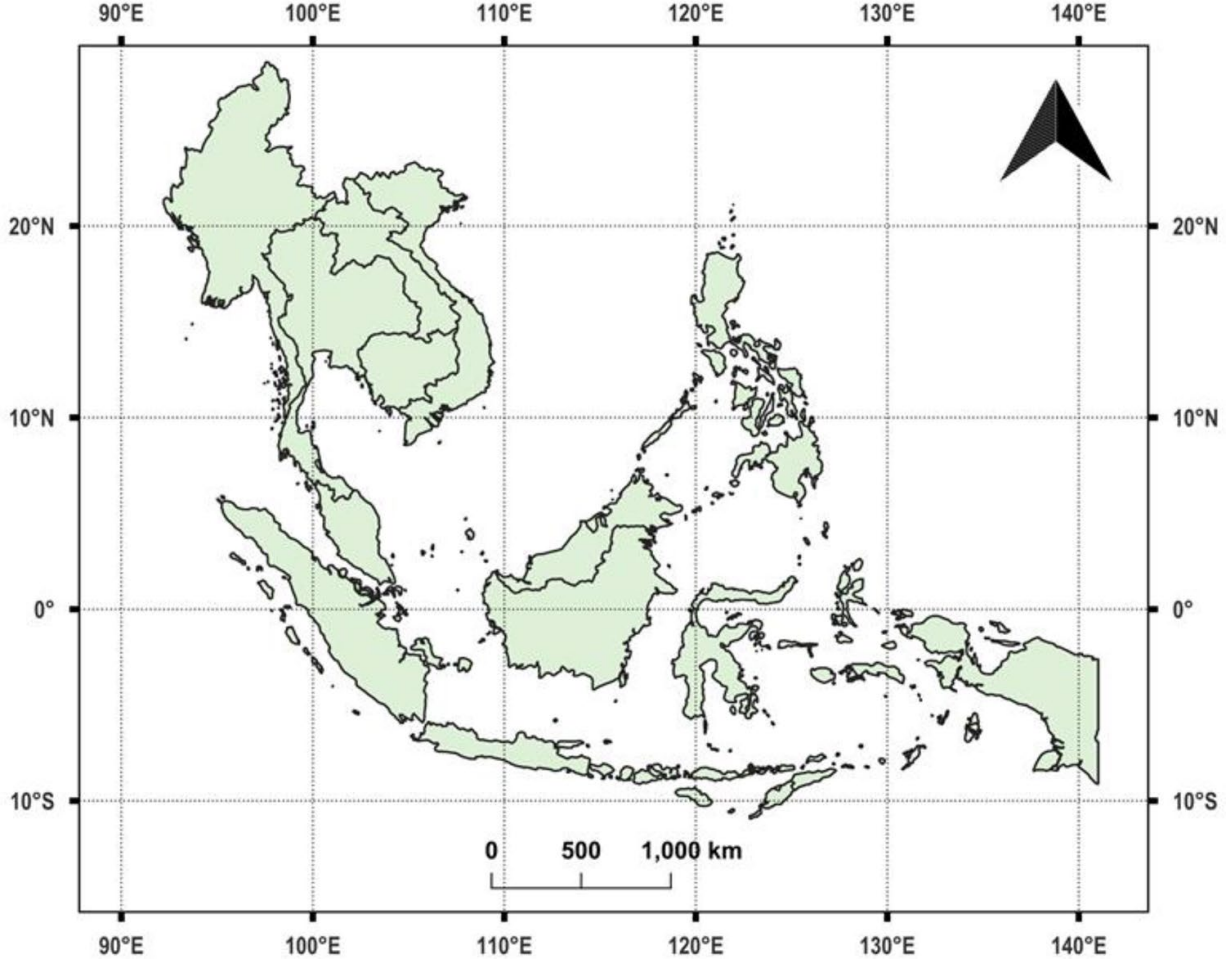
Study area covering Southeast Asia.

**Fig. 2:**
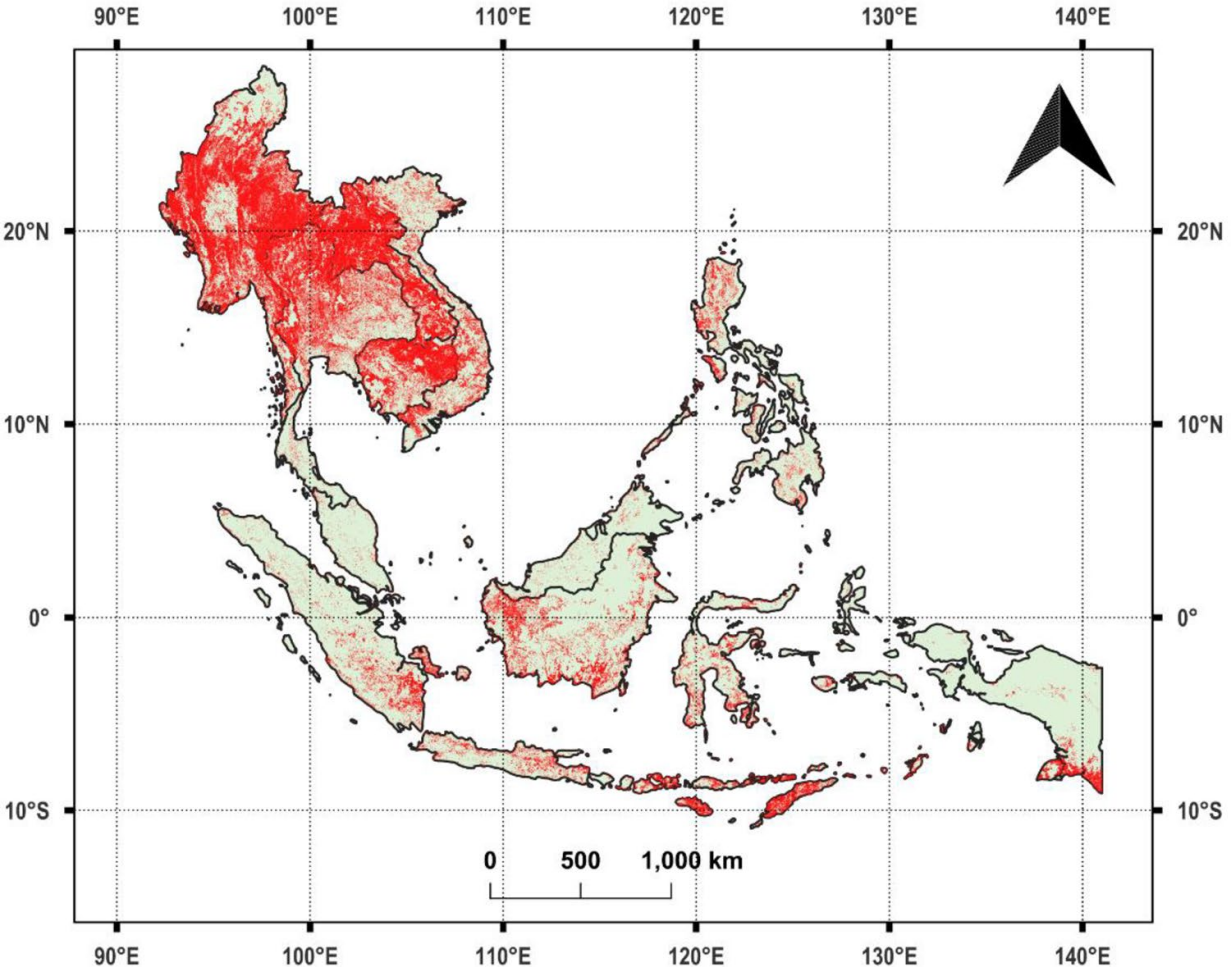
Fire occurrences (in red color) captured by the Visible Infrared Imaging Radiometer Suite (VIIRS) satellite in Southeast Asia during 2023.

**Fig. 3:**
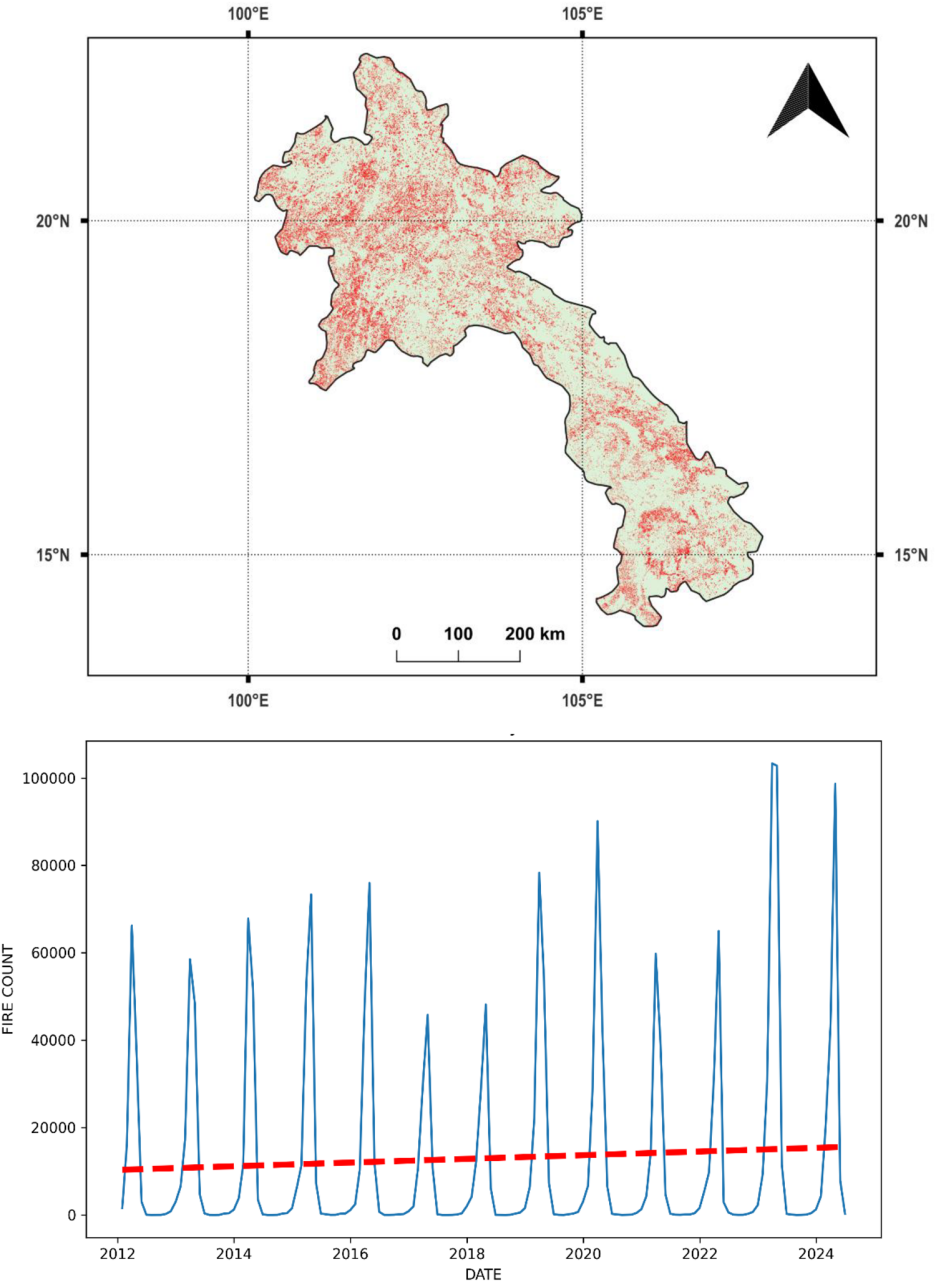
(**a**, **b**) Spatial patterns of vegetation fires derived from VIIRS satellite data in. The top figure (3a) shows the location and spatial patterns of vegetation fires (in red) derived from VIIRS satellite data in Laos. The bottom figure (3b) depicts fire count data for different years (2012–2024), indicating an increasing trend.

**Fig. 4:**
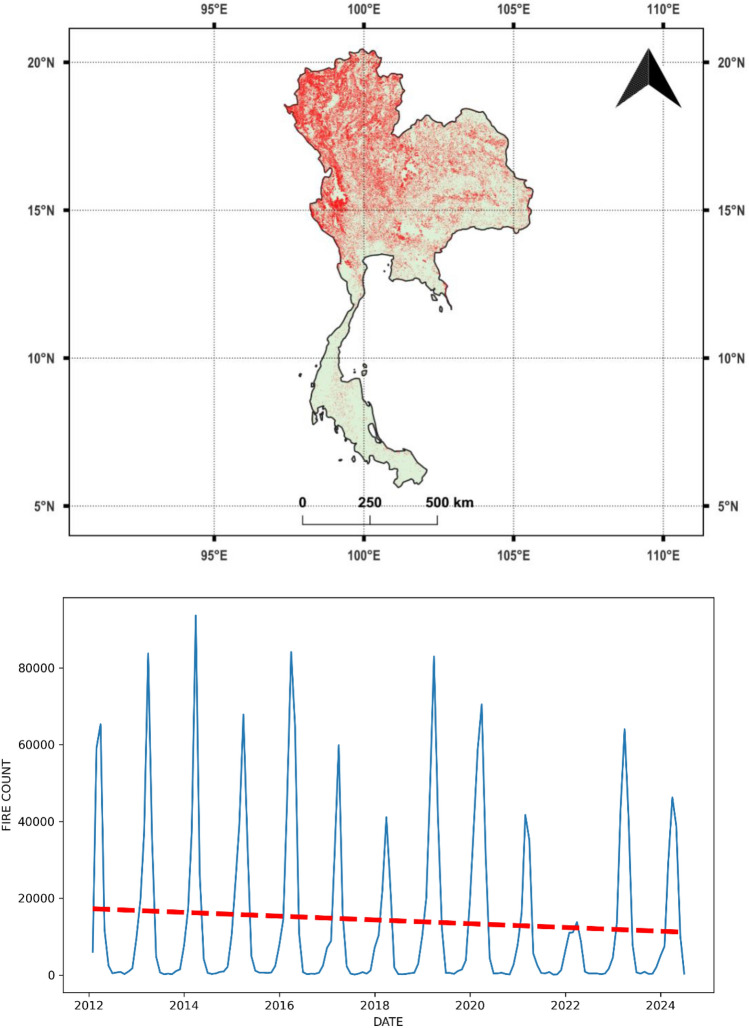
The top figure (4a) shows the location and spatial patterns of vegetation fires (in red) derived from VIIRS satellite data in Thailand. The bottom figure (4b) depicts fire count data for different years (2012–2024), indicating a decreasing trend.

We focused our study on Southeast Asian countries, which included Brunei, Cambodia, Indonesia, Laos, Malaysia, Myanmar, Philippines, Timor Leste, Thailand, and Vietnam (Fig. 1). Singapore was excluded due to its negligible fire occurrences. The selection of these countries was based on long-term fire activity trends and the availability of satellite fire data, ensuring the study remains relevant for fire prediction and mitigation efforts. Figure 2 illustrates the spatial distribution of fires across Southeast Asia in 2023, using VIIRS fire detections. The scattered red pixels represent fire occurrences, where higher concentrations of red indicate intense fire activity. The landscapes in this region are characterized by striking heterogeneity, with a mix of dense tropical rainforests, coastal mangroves, peatlands, grasslands, and mountainous areas. This region supports rich biodiversity but also challenges managing vegetation fires, which vary across the region due to differing land use, climatic patterns, and human activity. For example, peatland fires are particularly problematic in Indonesia and Malaysia, often exacerbated by agricultural clearing and drainage, leading to persistent and intense fires that release substantial carbon emissions. Cambodia, Laos, and Myanmar face seasonal fires that impact dry forests and grasslands, primarily driven by agricultural expansion and shifting cultivation practices. Meanwhile, the Philippines, Thailand, and Vietnam experience smaller-scale fires in forested areas and grasslands, often triggered by land conversion activities. Figures 3 and 4 provide a more detailed view of spatial fire patterns in Laos (2019) and Thailand (2020). Further, fires from crop residue burning are common in all the above countries.

Also, each country exhibits different temporal trends in fire activity. Figures 3b and 4b highlight fire seasonality and trends for Laos and Thailand, respectively. Figure 3b shows an increasing trend in Laos, indicating rising fire risks. In contrast, Fig. 4b demonstrates a decreasing trend in Thailand, suggesting improved fire management efforts or environmental factors reducing fire occurrences. These fires not only affect air quality and public health but also disrupt local ecosystems, with the complexity of the landscapes posing significant challenges for fire detection, management, and mitigation across the region. Efforts are underway to enhance fire management practices and promote sustainable agricultural techniques in these countries. However, challenges persist due to economic reliance on traditional farming practices and the costs associated with fire management and mitigation. Therefore, fire modeling studies can be crucial in addressing these issues and informing more effective fire management strategies.

## Datasets

We used the 375 m active fire product derived from the Visible Infrared Imaging Radiometer Suite (VIIRS), a key instrument aboard the Suomi National Polar-orbiting Partnership (Suomi NPP) and NOAA-20 satellites. VIIRS is designed to capture high-resolution optical and thermal data, enabling the detection of active fires globally in near real-time. The sensor operates across 22 spectral bands, including thermal infrared channels, allowing precise fire detection even in remote and cloud-covered regions. In contrast to other coarser resolution satellite fire detection products such as MODIS (≥ 1 km), the improved 375 m data provide increased detection of smaller fires and improved mapping of large fire perimeters^18^.

The VIIRS 375 m fire product builds on the earlier MODIS fire product heritage^19–21^, using a multi-spectral contextual algorithm to identify sub-pixel fire activity and other thermal anomalies in the Level 1 (swath) input data. The algorithm uses all five 375 m VIIRS channels to detect fires and separate land, water, and cloud pixels in the image^18^. The data is provided in various formats, including TXT, SHP, KML, and WMS, allowing easy integration into geospatial analysis platforms. Although the VIIRS fire data is well calibrated and validated globally, biases can occur due to cloud cover, smoke, and atmospheric conditions that obscure fire detection, leading to underreporting in certain regions. Temporal resolution limitations could result in missed, more minor, or short-lived fires^18^.

The latest VIIRS fire data are available from the University of Maryland’s fuoco SFTP server (sftp://fuoco.geog.umd.edu). A summary of the VIIRS dataset’s key attributes is provided in Appendix Table A1. This table outlines the primary characteristics, including spatial resolution, temporal frequency, and key fire-related data variables. We used the daily fire data and gridded to monthly ones at 5-arcminutes, i.e., 9.27 sq. km intervals before training the models. The dataset was formatted into sequential monthly time-series inputs, ensuring that models effectively captured temporal dependencies in fire occurrences. This study focuses on fire prediction based solely on historical fire occurrence data retrieved from VIIRS. No additional feature selection was applied to the original data. Our approach evaluates whether different machine and deep learning models can effectively capture patterns and trends in past fire data without incorporating external factors such as temperature, precipitation, or human activities. The models were trained and tested on original fire count data from VIIRS without additional predictor variables. This allows for an unbiased comparison of how well each model fits and forecasts fire occurrences based on historical trends. While environmental and anthropogenic factors play a crucial role in fire ignition and spread, this study does not attempt to model causal drivers. Instead, it focuses on short-term fire occurrence prediction based purely on past fire observations. Future research could explore additional drivers of fires for forecasting additional years.

## Methodology

We utilized the VIIRS fire datasets from 2012 to the present for our fire prediction studies, encompassing 150 months of data. First, we calculated the Mann–Kendall seasonal trend test on the fire data (2012–2024). The test is a non-parametric method for identifying trends in time series data. The test evaluates the existence of monotonic trends (increasing or decreasing) in a series without assuming any specific distribution. The results of this test are commonly presented in terms of the total Mann–Kendall Statistic (S), which measures the direction and strength of the trend, and the combined Z-statistic, which is a standardized version of the S-statistic used to assess statistical significance.

We applied a 72–28 data split, where 108 months (72%) were used for training and 42 months (28%) were reserved for testing. The training period spans from January 2012 to December 2020 (9 years), while the testing period covers January 2021 to June 2024 (3 and a half years). This structured allocation ensures that the models are trained on a sufficiently large dataset while being evaluated on more recent, unseen data to assess their predictive accuracy.

Given the sequential nature of fire data (a time series), time-based cross-validation was employed instead of traditional k-fold methods, which would disrupt temporal dependencies. Specifically, we implemented walk-forward validation, where the training window was progressively expanded before making each prediction. This approach ensures that models adapt to evolving fire trends while preventing data leakage from future observations. We implemented six different models to forecast fire occurrences, as described below. For each model, we plotted a fitted curve to visualize how well the model captured the fire patterns. Additionally, we included a trendline to highlight the overall direction and changes in fire occurrences over time.

The selection of ML/DL models in this study was based on their suitability for time-series forecasting and their ability to capture temporal dependencies in fire occurrences. The models include Persistence, MLP, CNN, LSTM, CNN-LSTM, and ConvLSTM, each offering different advantages in handling sequential data. While other models, such as Gated Recurrent Units (GRU) and Random Forest (RF), could also be explored, they were not included due to time constraints and computational feasibility.

### Simple persistence

This method served as an essential reference point. The Simple Persistence model assumes that future fire occurrences follow the same pattern as past data. Specifically, we used the median value of fire counts from previous seasonal timesteps (12-month intervals), such as 12, 24, and 36 months, to predict future values.

### Multilayer perceptron

Multilayer Perceptrons (MLPs) are an artificial neural network (ANN) designed to model complex nonlinear relationships between input and output variables. An MLP consists of an input layer, one or more hidden layers, and an output layer. Each layer comprises neurons, which process information by applying weights and biases to the inputs^22^. In an MLP, the output from the input layer is fed into the first hidden layer, where neurons compute weighted sums of their inputs and apply an activation function. This process continues through subsequent layers until the final output layer produces the model’s prediction. Training an MLP typically involves the back-propagation algorithm, which adjusts the weights based on the difference between predicted and actual outputs. The learning process is guided by a loss function, such as mean squared error, to optimize the model’s performance^23^. Important equations highlighting the MLP are given below:

1. *Input layer* The input to the network is represented as:
  $${\mathbf{x}} = [x_{1} ,x_{2} , \ldots ,x_{n} ]$$
  where n is the number of input features.
2. *Weighted sum* For a neuron *j* in the hidden layer, the weighted sum of inputs is given as,
  $$z_{j} = \sum\limits_{i = 1}^{n} {w_{ij} } x_{i} + b_{j}$$
  where *w*_*i*j_ is the weight connecting input x_i_ to neuron j, and b_j_ is the bias term for neuron *j*.
3. *Activation function* The output of the neuron after applying an activation function *f* (in this case, ReLU) is given as
  $$a_{j} = f(z_{j} )$$
4. *Output layer* For the output layer neuron ***K,***
  $$z_{k} = \sum\limits_{j = 1}^{m} {w_{jk} } a_{j} + b_{k}$$
  where m is the neuron in the previous layer.
5. Final output of MLP is given as,
  $$\hat{y} = f(z_{k} )$$
  where, $\hat{y}$ is the predicted output.
6. *Loss function* The mean squared error (MSE) for regression is given as,
  $$L = \frac{1}{N}\sum\limits_{i = 1}^{N} {(\hat{y}_{i} - y_{i} )^{2} }$$
  where, N is the number of samples and y_i_ is the target value.
7. *Back-propagation update rule* the weights are updated using,
  $$w_{ij} \leftarrow w_{ij} - \eta \frac{\partial L}{{\partial w_{ij} }}$$
  where, $\eta$ is the learning rate.

### Convolutional neural networks

The Convolutional Neural Network (CNN) algorithm is the most well-known and frequently used one in Deep Learning^24,25^. CNNs have 3 layers namely convolutional layer, pooling layer, and fully connected layer. The convolutional layer is a fundamental piece of the CNN architecture. It deals with performing feature extraction through a combination of linear and nonlinear functions, which are convolutional operations and activation functions. The pooling layer is fundamentally a down sampling operation where it reduces the features’ dimensionality and the number of learning parameters. The fully connected layer maps the extracted features into a final output^26^. Normalization and Standardization are two frequently used pre-processing techniques. Normalization helps in rescaling the data between 0 and 1 whereas standardization helps rescaling the value distribution so that the mean of the data is 0 and its standard deviation is 1^27^. Distinct equations for CNN operation on convolution and pooling are given below.

The convolution operation is given as,

$$Z_{j} = \sum\limits_{i = 1}^{k} {(W_{ij} *X_{i} )} + b_{j}$$

Pooling operation is given as,

$$P_{j} = {\text{pool}}(A_{j} )$$

### Long short-term memory (LSTM)

The Recurrent Neural Networks (RNNs) are superior in comparison to the traditional feed-forward networks^28^. LSTM is a member of the Recurrent Neural Networks (RNN) which have the functionality of vigorously incorporating historical experience due to internal recurrence. These networks can remember information and connect the data obtained in the past to the present^29^. LSTM has a memory of its own, and so it is used in making accurate forecasting and prediction scenarios 30; thus, it is widely used in speech recognition, text analysis, language modeling, and stock market forecasting^30,31^. Conventional RNNs face significant challenges with gradient explosion and gradient disappearance. Gradient explosion occurs when gradients become excessively large during backpropagation, leading to unstable weight updates and erratic training. Conversely, gradient disappearance happens when gradients become too small, making it difficult for the network to learn long-range dependencies in sequential data. These issues limit the effectiveness of traditional RNNs, prompting the development of advanced architectures like Long Short-Term Memory (LSTM) networks and Gated Recurrent Units (GRUs), which better manage gradient flow and enhance learning from sequences^32^.

The LSTM network is similar to the typical RNNs with the exception of the introduction of three gates, namely input gates, output gates, and forget gates, in the memory cell, which controls the flow direction of information within the network^27^. These cells can be thought of as performing read, write, and reset actions on a cell state^33^. The following equations define the workings of the LSTM model.

1. *Forget gate* it determines which information to discard from the cell state. It uses the sigmoid activation function to output values between 0 and 1, where 0 means “forget” and 1 means "keep."
  $$f_{t} = \sigma (W_{f} \cdot [h_{t - 1} ,x_{t} ] + b_{f} )$$
2. *Input gate* it decides what new information to store in the cell state. It uses the sigmoid function as the forget gate to produce values for the input update.
  $$i_{t} = \sigma (W_{i} \cdot [h_{t - 1} ,x_{t} ] + b_{i} )$$
3. *Cell State update* Creates a vector of new candidate values to add to the cell state, ensuring the values are between -1 and 1 using the tanh function.
  $$\tilde{C}_{t} = \tanh (W_{C} \cdot [h_{t - 1} ,x_{t} ] + b_{C} )$$
4. *Update Cell state* This process combines the old and new candidate values moderated by the forget and input gates to create the updated cell state.
  $$C_{t} = f_{t} \cdot C_{t - 1} + i_{t} \cdot \tilde{C}_{t}$$
5. *Output gate* it determines, what part of the cell state to output. It also uses the sigmoid function to control the influence of the cell state on the hidden state.
  $$o_{t} = \sigma (W_{o} \cdot [h_{t - 1} ,x_{t} ] + b_{o} )$$
6. *Hidden State update* This step involves the calculation of the hidden state for the current time step, using the output gate and the cell state to determine the final output for the LSTM unit.
  $$h_{t} = o_{t} \cdot \tanh (C_{t} )$$

The brief description of each variable in the above equations are as follows: f_t_ is the forget gate output which controls what information to discard from the cell state; σ is the sigmoid activation function with values from 0,1; W_f_ is the weight matrix for the forget gate; h_t-1_ is the hidden state from the previous time step; x_t_ is the input vector at the current time step; b_f_ is the bias vector for the forget gate; i_t_ is the input gate output which determines which new information to store in the cell state; W_i_ is the weight matrix for the input gate; b_i_ is the bias vector for the input gate; C_t_ is the candidate cell state, representing new information to be added; W_C_ is the weight matrix for the candidate cell state; b_C_ is the bias vector for the candidate cell state; C_t_ is the updated cell state, which carries long-term memory; o_t_ is the output gate output which controls what part of the cell state is sent to the hidden state; W_o_ is the weight matrix for the output gate; b_o_ is the bias vector for the output gate and h_t_ is the hidden state at the current time step, which is the output of the LSTM cell.

### CNN-LSTM

This deep learning model combines both CNN and LSTM models. To significantly detect and model the short- and long-term temporal interrelationships cornified in the dataset order, a powerful combination of CNN-LSTM (Convolutional Neural Network-Long Short-Term Memory) has been proposed^34^. The CNN acts as an encoder, and the LSTM serves as a decoder. A feature is learned by the encoder from the input data and supplied to the decoder. After that, the decoder recognizes and models any intrinsic short- and long-term temporal relationships in the dataset^34^. To analyze the performance of this deep learning model, Mean Squared Error (MSE) was used. Important equations specific to CNN-LSTM architecture are given below:

1. *Convolution operation* this represents the convolution operation applied to the input data, where W_ij_ is the filter or kernel weights, and X_i_ are the input features.
  $$Z_{j} = \sum\limits_{i = 1}^{k} {(W_{ij} *X_{i} )} + b_{j}$$.
2. *Pooling operation* It represents the pooling layer, which reduces the spatial dimensions of the feature maps produced by the convolution operation and given as,
  $$P_{j} = {\text{pool}}(A_{j} )$$
3. *Flattening* this operation converts the pooled feature into a one-dimensional vector to be fed into the LSTM layer.
  $$F = {\text{flatten}} (P_{j} )$$
4. *LSTM output* The equation below represents the LSTM processing the flattened feature vector F, the previous hidden state ht-1, and cell state C_t−1_ to produce the current hidden state.

The brief description of each variable in the above equations is as follows: Zj​: is the output of the convolution operation for filter j. It represents the feature output generated by applying a convolutional filter to the input; Wij is the weights of the convolutional filter j, which are learned during the training process; Xi is the input features at position i, which refer to the data being convolved (time series in our case), Pj is the output of the pooling operation for feature j; Aj represents activation from the convolution layer and is the result of after applying the activation function to the convolution output, and F represents flattened vector from the pooled feature and is the one-dimensional representation of the feature maps, prepared for the LSTM layer.

### Convolutional LSTM (ConvLSTM) ConvLSTM

ConvLSTM brings the benefits of both CNN and LSTM together^35,36^. It has the architecture of LSTM embedded into convolution^37^. ConvLSTM is a kind of recurrent neural network used for spatiotemporal prediction that incorporates convolutional structures in both input-to-state and state-to-state transitions. By using the inputs and previous states of its local neighbors, the ConvLSTM predicts the future state of each cell in the grid. A convolution operator in the state-to-state and input-to-state transitions makes this simple to accomplish^38^. In ConvLSTM, the method primarily focuses on the integration of convolution operations within the LSTM framework. Each gate uses convolution operation instead of standard matrix multiplication for inputs and hidden states. The equations for the forget, input, candidate cell state and output gates are as follows:

1. Forget gate:
  $$f_{t} = \sigma (W_{f} *X_{t} + U_{f} *h_{t - 1} + b_{f} )$$
2. Input gate:
  $$i_{t} = \sigma (W_{i} *X_{t} + U_{i} *h_{t - 1} + b_{i} )$$
3. Candidate cell State
  $$\tilde{C}_{t} = \tanh (W_{C} *X_{t} + U_{C} *h_{t - 1} + b_{C} )$$
4. Output gate
  $$o_{t} = \sigma (W_{o} *X_{t} + U_{o} *h_{t - 1} + b_{o} )$$

The formulas for updating the cell and hidden states remain like LSTM, as they use the outputs of the gates defined above. The overall distinction in ConvLSTM is the use of convolutional operations to capture spatial features in the input data, making it suitable for tasks involving spatial–temporal data or spatiotemporal forecasting, as in our fire-temporal data in this study.

We tested these models for the VIIRS satellite derived fire datasets for individual countries in South/Southeast Asia. For the model evaluation, we used the root mean square error (RMSE), which is a commonly used metric to measure the accuracy of a model’s predictions compared to actual observed values. RMSE is a single number that indicates how close the predicted values are to the observed data, with lower values representing better model performance. RMSE is given as,

$$RMSE = \sqrt {\frac{1}{n}\sum\limits_{i = 1}^{n} {(\hat{y}_{i} - y_{i} )^{2} } }$$

where n is the number of observations, $\widehat{{\text{y}}}$_*i*_ the predicted value for observation, y_i_ is the is the actual observed value for observation i.

### Hyperparameter optimization

We performed a grid-search based hyperparameter optimization for all models and for all countries using the training period 2012 to 2020. Depending on the architecture of the model, various parameters were included such as input size, number of nodes, number of epochs, number of filters, kernel size, batch size, step size, and sequence size. The Persistence model, used as a baseline, did not require any tuning. For MLP, the parameters optimized were input size, number of nodes, epochs, and batch size. CNN tuned parameters were input size, filters, kernels, epochs, and batch size. LSTM was tuned for input size, nodes, epochs, batch size, and difference. For CNN-LSTM and ConvLSTM models step size, sequence size, number of filters, kernel size, epochs, and batch size were tuned. Each model was optimized independently for each country. A more detailed summary of hyper parameter range is provided in the Appendix Table A2.

Given the variation in time series patterns across countries, model configurations were selected independently for each case. For every model-country combination, the parameter set yielding the lowest RMSE on the training data was chosen, ensuring tailored optimization while avoiding data leakage into the test period. It must be noted that the models could be tuned more finely by exploring additional combinations or narrower parameter ranges, such exhaustive searches were limited by available computational resources and time.

## Results and discussion

**Fig. 5:**
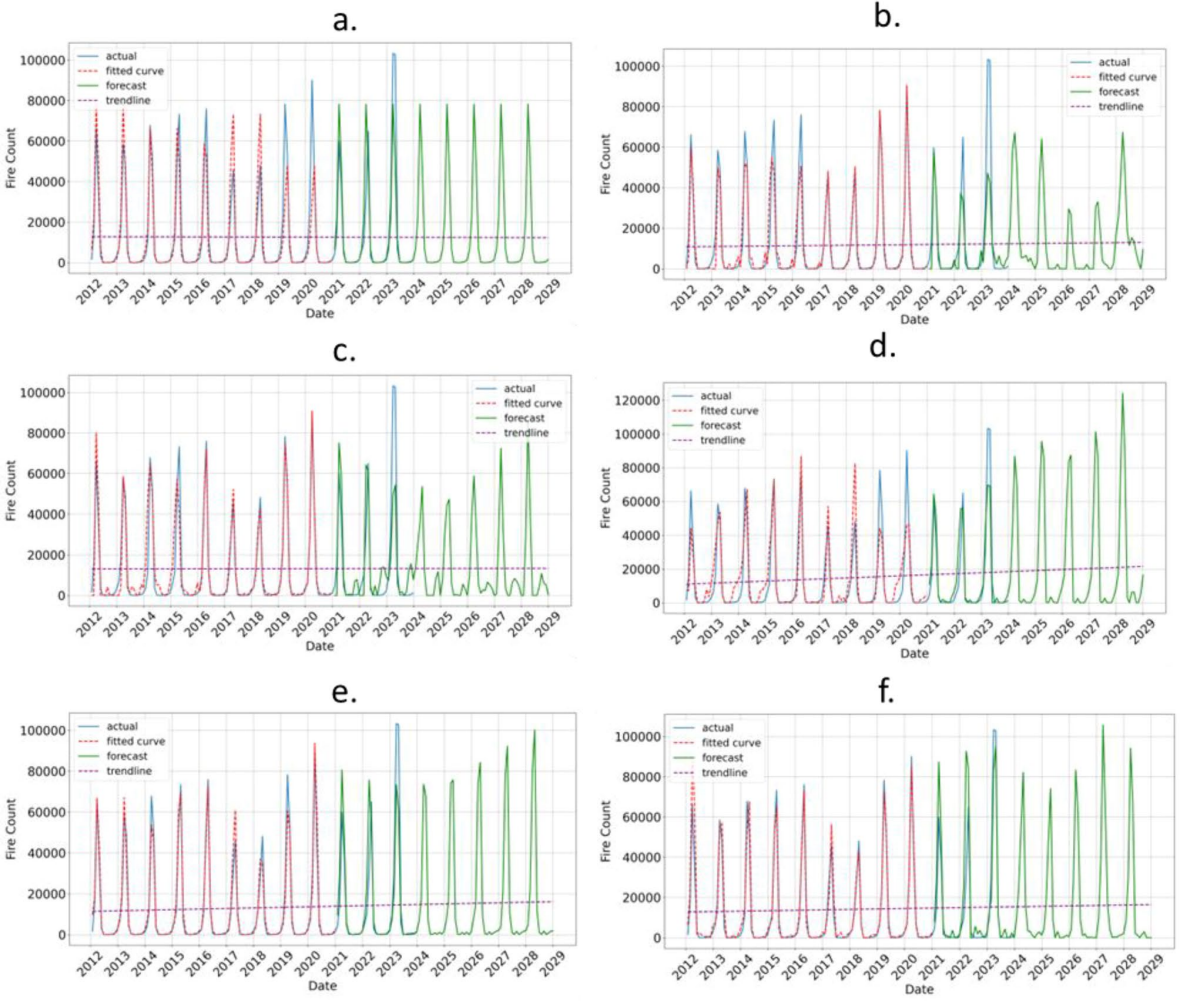
(**a**–**f**) Fire prediction in Laos using six different models: (**a**) Persistence, (**b**) MLP, (**c**) CNN, (**d**) LSTM, (**e**) CNN-LSTM, and (**f**) ConvLSTM. The VIIRS satellite derived fire data is shown in blue (2012–2023), while the fitted model curve is represented in red (2012–2020). The fire forecast is displayed in green (2021–2029), with the trend line shown as a dotted purple line. Among the various models, ConvLSTM, which combines convolutional and LSTM layers, achieved the lowest RMSE in Laos, indicating a potential need to integrate both spatial and temporal patterns for fire prediction in this country.

**Fig. 6:**
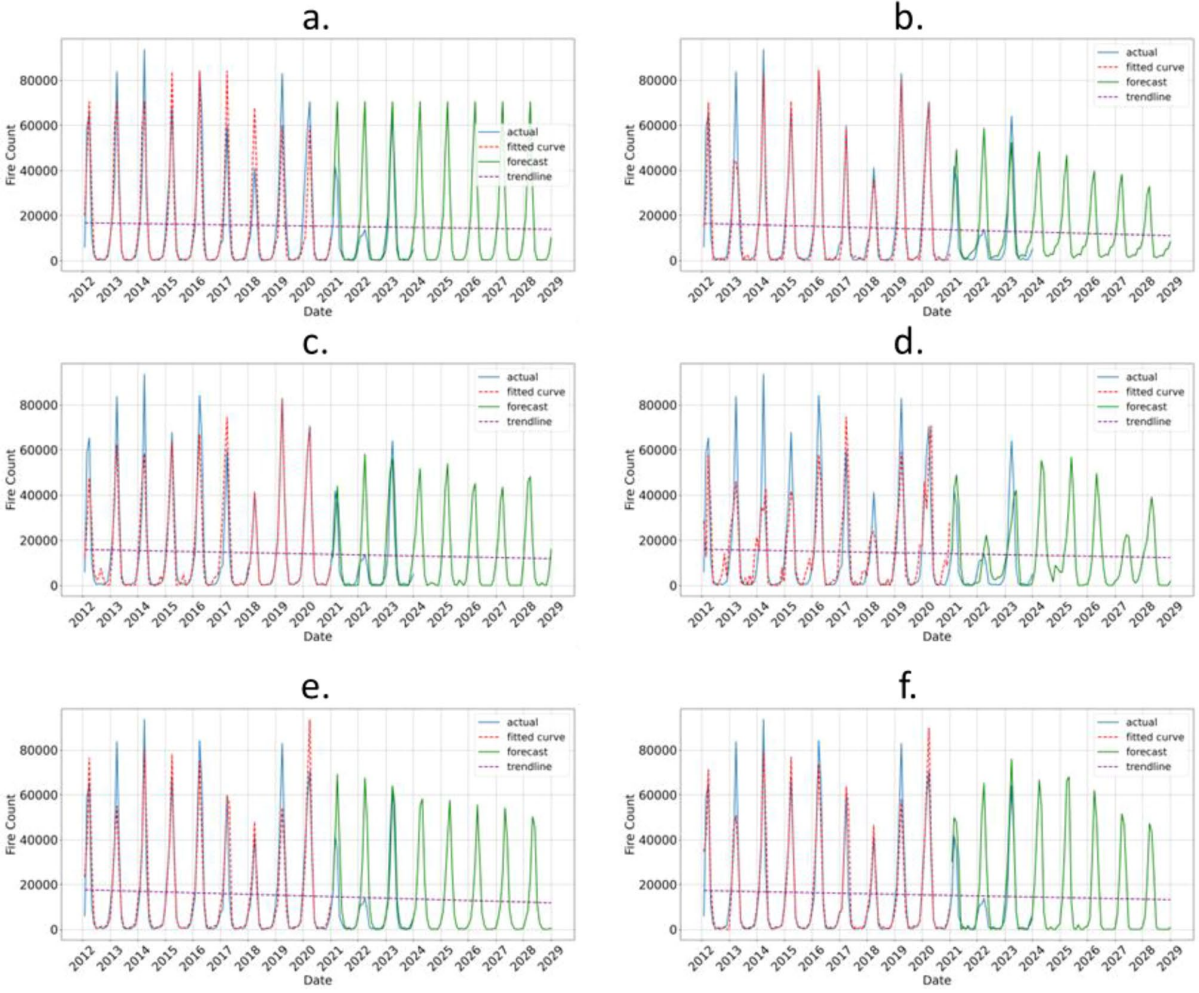
(**a**–**f**) Fire prediction in Thailand using six different models: (**a**) Persistence, b. MLP, c. CNN, (**d**) LSTM, (**e**) CNN-LSTM, and (**f**) ConvLSTM. The VIIRS satellite-derived fire data is shown in blue (2012–2023), while the fitted model curve is represented in red (2012–2020). The fire forecast is displayed in green (2021–2029), with the trend line shown as a dotted purple line. Among different models, the CNN model has the lowest RMSE in Thailand, suggesting that a convolutional approach captures the relevant patterns in the data better than other models.

The fire prediction results for sample data from Laos and Thailand are illustrated in Figs. 5a–f and 6a–f, respectively. The results for other Southeast Asia countries are given in Appendix (Figures S1–S3). For all countries, including Thailand and Laos, six models were utilized for fire prediction: (a) Persistence, (b) MLP, (c) CNN, (d) LSTM, (e) CNN-LSTM, and (f) ConvLSTM. The fire data collected from the VIIRS satellite is represented in blue for 2012 to 2023, with the fitted model curve shown in red for 2012 to 2020. Forecasts for the years 2021 to 2029 are displayed in green, while a dotted purple line indicates the trend.

Before the model evaluations, we also assessed the temporal fire trends from 2012 to 2014 using the Mann–Kendall seasonal trend statistic for the various countries. Results suggested Brunei, Cambodia, and Indonesia all show negative Mann–Kendall Statistics, indicating a slight decline in their respective datasets. However, the Z-statistics for these countries are relatively close to zero, with values of − 0.1773 for Brunei, − 0.3949 for Cambodia, and − 0.5037 for Indonesia, which indicates that despite the observed decrease in these countries, the trends are not strong. In contrast, Laos, Myanmar, the Philippines, and Timor Leste all exhibit weak positive trends, with total Mann–Kendall Statistics of 125, 59, 32, and − 131, respectively. Laos, which has a positive statistic, also shows a Z-statistic of 0.2031. Myanmar and the Philippines display Z-statistics close to zero (0.0959 and 0.0520, respectively), implying that their slight increases are insignificant. Despite showing a negative statistic, Timor Leste also failed to show statistical significance with a Z-statistic of − 0.2129. Malaysia and Thailand are more comparable to the first group of countries with negative Mann–Kendall Statistics of − 315 and − 118, respectively, suggesting a declining trend and a Z-statistic of − 511 and − 0.1917, respectively. Similarly, Vietnam, with a Mann–Kendall Statistic of − 122 and a Z-statistic of − 0.1982, also reflects a weak, statistically insignificant decrease. In comparing and contrasting these countries, the overall picture is that most countries showed an increasing or decreasing trend, with less statistically significant seasonal trends. It is important to note that the model parameterization and fitting were performed based on the original data without any modifications.

The ConvLSTM model, which integrates convolutional and LSTM layers, demonstrated the lowest RMSE for fire prediction in Laos, highlighting the necessity of incorporating both spatial and temporal patterns in this context. In contrast, the CNN model achieved the lowest RMSE in Thailand, indicating that a convolutional approach is more effective at capturing relevant data patterns compared to other models.

Tables 1, 2, and 3 present the evaluation metrics used for model comparison across different Southeast Asian countries. Table 1 provides Root Mean Square Error (RMSE) values, Table 2 presents Mean Absolute Error (MAE), and Table 3 lists R-squared (R^2^) values. These tables highlight the best-performing model for each region, with the lowest error values marked in bold for RMSE and MAE tables, and highest R-squared values.

**Table 1:** Root Mean Square Error (RMSE) for fire prediction models.

Country	Persistence	MLP	CNN	LSTM	CNN-LSTM	ConvLSTM
Brunei	12.94	10.26	**9.32**	11.04	17.08	11.21
Cambodia	12,670.55	11,727.27	10,837.76	13,740.28	**9784.36**	10,278.68
Indonesia	15,109.57	13,968.02	**7278.05**	13,705.47	14,816.29	14,816.29
Laos	15,925.18	16,933.19	14,309.07	16,299.57	15,827.33	**13,241.27**
Malaysia	708.30	580.95	**416.75**	633.69	509.51	493.62
Myanmar	18,234.55	16,331.57	15,532.29	48,590.99	17,117.65	**13,965.56**
Philippines	3046.91	1837.61	**1666.81**	2938.77	2275.95	2394.37
Timor-Leste	554.76	484.81	**400.40**	504.52	443.27	529.91
Thailand	14,315.45	15,966.57	**11,387.51**	14,262.14	15,890.23	15,519.61
Vietnam	2962.91	2953.22	2422.58	3346.78	2265.44	**2207.06**

**Table 2:** Mean Absolute Error (MAE) for fire prediction models.

Country	Persistence	MLP	CNN	LSTM	CNN-LSTM	ConvLSTM
Brunei	7.405	6.381	**6.21**	6.333	9.762	6.476
Cambodia	7142.98	6694.86	6935.43	9654.69	**5264.95**	6761.81
Indonesia	8202.69	13,841.12	**7673.21**	7693.79	7885.14	9066.95
Laos	7384.02	8748.55	8468.02	7796.02	7517.98	**6586.36**
Malaysia	490.91	449.81	**323.64**	477.81	384.21	344.62
Myanmar	9393.17	8474.17	9659.76	27,164.83	9241.93	**8181.05**
Philippines	1732.55	1251.76	**1066.12**	1814.81	1251.43	1528.76
Timor-Leste	243.86	218.74	**175.24**	229.81	254.29	250.10
Thailand	7694.29	8121.48	**7213.79**	7772.21	8096.12	9111.48
Vietnam	1762.50	1999.02	1810.50	2384.21	1566.48	**1418.07**

**Table 3:** R^2^ values for fire prediction models.

Country	Persistence	MLP	CNN	LSTM	CNN-LSTM	ConvLSTM
Brunei	− 0.57	0.01	**0.19**	− 0.14	− 1.74	− 0.18
Cambodia	0.50	0.57	0.63	0.41	**0.70**	0.67
Indonesia	0.12	0.26	**0.80**	0.28	0.15	0.25
Laos	0.69	0.65	0.75	0.67	0.69	**0.79**
Malaysia	− 1.15	− 0.45	**0.26**	− 0.72	− 0.11	− 0.05
Myanmar	0.87	0.89	0.90	0.06	0.88	**0.92**
Philippines	− 0.03	0.63	**0.69**	0.04	0.42	0.36
Timor-Leste	− 0.08	0.17	**0.44**	0.10	0.31	0.01
Thailand	0.22	0.03	**0.51**	0.23	0.04	0.09
Vietnam	0.58	0.58	0.72	0.46	0.75	**0.76**

Specific to RMSE, the metric showed higher values which can be attributed to highly varying spatial and temporal nature of fires in the region and inherent fire data characteristics used from 2012 to 2024. The Convolutional Neural Network (CNN) model demonstrated consistently strong performance across Brunei, Indonesia, Malaysia, the Philippines, Timor-Leste, and Thailand. In these countries, CNN achieves the lowest RMSE values, with standout performances of 9.32 in Brunei, 7278.05 in Indonesia, 416.75 in Malaysia, 1666.81 in the Philippines, 400.40 in Timor-Leste, and 11,387.51 in Thailand. This suggests that CNN effectively captures spatial patterns relevant to the metrics for these regions. Meanwhile, the ConvLSTM model shows the best results for Laos, Myanmar, and Vietnam, suggesting it is more effective in areas with complex spatiotemporal dynamics. ConvLSTM achieves an RMSE of 13,241.27 in Laos, 13,965.56 in Myanmar, and 2207.06 in Vietnam. For Cambodia, the CNN-LSTM hybrid model stands out, performing best with an RMSE of 9784.36, indicating that the combined spatial and temporal features captured by CNN-LSTM suit the data characteristics in this region. Overall, CNN is the most frequently optimal model for regions with predominantly spatial dependencies, while ConvLSTM and CNN-LSTM excel in areas requiring a blend of spatial and temporal feature extraction. Model specific results and important discussion points are provided below.

The Persistence model, which assumes future values mirror past observations, serves as a simple and computationally efficient baseline, occasionally capturing patterns in stable or slow-changing phenomena. However, its limitations are evident in its high RMSE values across most countries, suggesting its ineffectiveness in capturing dynamic or rapidly changing systems, such as those influenced by urbanization or climate variability. In countries like Indonesia, Myanmar, and Laos, the Persistence model performs poorly, with RMSE values significantly higher than those of more advanced models, highlighting its inability to account for complex trends and temporal patterns.

The MLP model, a basic neural network architecture, generally outperformed the Persistence model, achieving relatively low RMSE values in countries with simpler or less dynamic patterns, such as Malaysia and Timor-Leste. However, its lack of mechanisms for handling temporal dependencies limited its effectiveness for sequential data that requires memory of previous time steps. Thus, in countries with complex temporal patterns, such as Laos and Myanmar, the MLP model’s RMSE values are considerably higher than those of CNN, LSTM, or ConvLSTM, underscoring its limitations in capturing intricate temporal dynamics and spatial dependencies within the data.

Compared to the persistence and MLP models, the CNN models are known for its ability to capture spatial dependencies. Thus, it demonstrated a strong performance across several countries, achieving the lowest RMSE values in Brunei (9.41), Indonesia (7278.05), Malaysia (416.75), the Philippines (1666.81), Timor-Leste (400.40), and Thailand (11,387.51). This indicates that CNNs are well-suited for capturing spatial patterns, making them effective in regions where spatial features, such as landscape characteristics, are significant predictors of fires. However, CNNs lack an inherent temporal memory, limiting their effectiveness for capturing time-dependent processes like long-term climatic trends or seasonal changes. This limitation is evident in countries like Myanmar and Laos, where higher RMSE values suggest that CNNs may struggle with complex temporal dynamics.

Relatively, LSTM models are well-suited for sequential data, capturing long-term dependencies and temporal trends, making them beneficial for datasets with strong temporal dynamics. Thus, we noted that in countries like Brunei and Malaysia, the LSTM model showed reasonable performance, though it did not reach the lowest RMSE values. However, LSTMs lack the ability to capture spatial dependencies, which may contribute to their relatively high RMSE values in regions like Cambodia, Laos, and Thailand, where both spatial and temporal factors are crucial. Additionally, LSTMs are computationally more demanding than simpler models, posing a potential drawback for large-scale or real-time applications.

In contrast to either CNN or the LSTM models alone, the CNN-LSTM hybrid model leverages both the spatial feature extraction of CNNs and the temporal memory of LSTMs, making it highly effective for spatiotemporal data. Thus, it performs particularly well in Cambodia, achieving the lowest RMSE (9784.36), indicating its strength in capturing both spatial and temporal patterns, especially useful for complex phenomena like agriculture or climate-driven processes. However, the CNN-LSTM model did not consistently outperform simpler models across all countries such as in Brunei, Indonesia, and the Philippines, CNN alone achieves better results, suggesting that the hybrid model’s complexity may be unnecessary in cases dominated by spatial patterns. Additionally, the CNN-LSTM model has higher computational requirements, which can limit efficiency compared to single-architecture models like CNN or LSTM.

ConvLSTM is an advanced model that integrates convolutional and recurrent layers to effectively capture spatial dependencies and maintain temporal memory in spatiotemporal data. Thus, it effectively captured spatial dependencies and temporal memory, achieving the lowest RMSE values in Laos (13,241.27), Myanmar (13,965.56), and Vietnam (2207.06). This makes it suitable for scenarios with complex spatiotemporal dependencies. However, its higher computational cost can be a limitation, particularly in regions like Brunei and Malaysia, where simpler spatial dependencies prevail. In these cases, CNNs or other basic models can outperform ConvLSTM, suggesting that its complexity may be unnecessary when temporal variability is lower, allowing simpler models to achieve comparable or better results with reduced resource demands.

The RMSE differences between models stem from their ability to capture temporal fire patterns. Simpler models like Persistence and MLP lack trend-learning capabilities, leading to higher RMSE values. Persistence assumes fire occurrences repeat past patterns, missing trends, and sudden changes. MLP offers slight improvements but struggles with sequential dependencies. CNN models, though designed for spatial data, effectively captured short-term temporal patterns, making them well-suited for fire prediction. ConvLSTM builds on this by combining spatial and temporal tracking, allowing it to model both short-term fluctuations and long-term trends. In contrast, LSTM, which specializes in long-term dependencies, performed worse.

In addition to RMSE, we evaluated model performance using MAE and R^2^ to ensure a more comprehensive assessment. R^2^ provides insight into how well the model explains variance in fire occurrence, while MAE offers an intuitive measure of prediction error by quantifying absolute deviations. The comparison of RMSE, MAE, and R^2^ across models further validates CNN and ConvLSTM as the best-performing models.

The results indicate that ConvLSTM achieved the highest R^2^ values and the lowest MAE, reinforcing its effectiveness in capturing fire trends. CNN also exhibited strong performance across all metrics, making it a competitive alternative. In contrast, simpler models such as Persistence and MLP showed lower R^2^ values and higher MAE, confirming their limitations in modeling fire occurrences.

ConvLSTM had the highest computational cost among the six models evaluated due to its combination of convolutional layers and LSTM units, which require more parameters and complex operations. This resulted in longer training times and higher memory usage, making real-time applications more challenging. In general, computational speed across models followed the pattern: Persistence < MLP < CNN < LSTM < CNN-LSTM < ConvLSTM. Hyperparameter tuning involved 5–7 parameters per model, each with multiple possible values, leading to a large configuration space particularly for deep architectures like CNN-LSTM and ConvLSTM. To manage this, we employed parallel processing and CPU acceleration using all available cores on the local system. Models designed for handling sequential dependencies, such as CNN-LSTM and ConvLSTM, require significantly more computational resources than simpler architectures like CNN and MLP. While ConvLSTM achieved better accuracy in some regions, its deployment in real-world settings would require hardware acceleration (e.g., GPUs/TPUs) and optimization techniques such as model pruning and quantization to improve efficiency. In contrast, simpler models like CNN and MLP are computationally lighter and can be more practical for operational use where rapid temporal predictions are required. Future research should explore the trade-off between model complexity and computational efficiency, balancing accuracy with real-time feasibility.

In summary, the CNN model is most effective in countries with fire data that had primarily spatial dependencies, such as Brunei, Indonesia, Malaysia, the Philippines, and Timor-Leste. Meanwhile, ConvLSTM performed well in countries with significant spatiotemporal dependencies, such as Laos, Myanmar, Thailand, and Vietnam. Cambodia is best served by the CNN-LSTM hybrid model, suggesting a need for balanced spatial and temporal processing capabilities. This pattern highlights the diverse data characteristics across Southeast Asian countries and the importance of selecting models suited to each region’s needs.

The insights gained from our analysis of satellite-derived fire data emphasize the critical need for model evaluation before integrating them into any decision support systems (DSS) for fire management and mitigation. Given the unique temporal and spatial patterns across Southeast Asian countries, a one-size-fits-all approach is insufficient; instead, tailoring models to each region’s specific characteristics is essential for effective decision-making^39^. Evaluating multiple machine learning and deep learning models upfront optimizes resource allocation by identifying the most efficient option and enhances the accuracy and reliability of predictions that inform critical management decisions. Incorporating both spatial and temporal patterns alongside historical trends has been shown to improve prediction accuracy and using robust statistical metrics^40,41^. Integrating changes in weather and recurring seasonal weather patterns can improve fire forecasting accuracy. Integrating socioeconomic factors into fire forecasting can improve accuracy by accounting for human behavior, land use changes, and infrastructure influencing fire risk, such as population density, urbanization^41^, and economic activities. Combining these factors with ensemble methods, which combine multiple models or algorithms, can improve prediction robustness^42,43^, particularly when addressing complex fire dynamics across varied regions. Ultimately, such a rigorous evaluation process can help improve and adapt models, ensuring that decision support systems remain responsive to fire-related risks and environmental challenges.

It should also be noted that the above deep learning models can be computationally intensive and require resources. For example, models such as ConvLSTM are computationally intensive due to their combination of convolutional operations for spatial feature extraction and LSTMs for temporal dependencies. Their high memory, storage, and processing demands make real-time applications challenging, often requiring Graphics Processing Units (GPUs), Tensor Processing Units (TPUs), or cloud-based solutions. Running typical deep learning models requires hardware, typically GPUs (e.g., NVIDIA A100, RTX 4090) for efficient training and inference or TPUs (e.g., Google TPUv4) for TensorFlow workloads. CPUs (e.g., Intel Xeon) are suitable for lightweight tasks. Cloud solutions like AWS, Google Cloud, Google Colab and Azure provide scalable GPU/TPU instances. A minimum of 16GB RAM is needed, with 32GB + for larger models, and SSDs are preferred for fast data access. Large input data and sequential dependencies increase inference time, leading to latency issues in time-sensitive applications. Additionally, high energy consumption can limit deployment on devices. Strategies like model pruning, quantization, parallel processing, and hybrid approaches can be employed to optimize real-time performance. Leveraging cloud computing can enhance efficiency, making these models more practical for real-time fire prediction and monitoring.

By customizing models to match each country’s fire patterns, authorities can better assess risks, use resources wisely, and take early action to reduce fire damage. The findings also show that these models must improve as fire patterns change. Future research should look at how factors such as land use, human activity, FRP, and location affect fires to make predictions more useful for prevention and response. Combining different modeling methods like deep learning with traditional approaches could make forecasts more accurate and reliable. Improving model efficiency would also help fire agencies get real-time updates, making fire prediction systems more practical for quick action. Using satellite data and new technology will be key to building more innovative and more adaptable fire management strategies.

## Conclusion

Our results on satellite derived fire data and evaluation of different machine learning and deep learning models across various Southeast Asian countries revealed significant insights into model performance relative to the underlying data characteristics of each country. The Convolutional Neural Network (CNN) model consistently emerged as the best performer in countries with predominantly spatial dependencies, such as Brunei, Indonesia, Malaysia, the Philippines, Timor-Leste, and Thailand. Conversely, the ConvLSTM model demonstrated superior performance in countries with intricate spatiotemporal dynamics in fire data, such as Laos, Myanmar, and Vietnam. This model’s architecture effectively integrates spatial and temporal data, essential in environments influenced by complex interactions over time. In contrast, in Cambodia CNN-LSTM hybrid model excelled, highlighting the region’s requirement to balance spatial and temporal features. This suggests that integrating both capabilities is necessary to model the dynamics of complex phenomena accurately. Our analysis highlights the need for rigorous model evaluation before integrating them into fire management decision support systems. Tailoring models to each country’s unique fire patterns can enhance risk assessment, resource allocation, and early intervention.

## Supplementary Information

Supplementary Information.

## Data Availability

The datasets used and/or analyzed during the current study available from the corresponding author on reasonable request.
