# Supplementary material for: Evaluation of machine learning and deep learning algorithms for fire prediction in Southeast Asia
Source: Sci Rep. 2025 May 29;15:18807. doi: 10.1038/s41598-025-00628-9 (PMC12122932; doi:10.1038/s41598-025-00628-9)

**Evaluation of Machine Learning and Deep Learning Algorithms for Fire Prediction in Southeast Asia**

Aditya Eaturu^1^ and Krishna Prasad Vadrevu^2^

^1^University of Alabama, Huntsville

^2^NASA Marshall Space Flight Center, Huntsville, Alabama, USA

**Corresponding author email: Krishna.p.vadrevu@nasa.gov**

**Appendix**

Table A1. VIIRS active fire data source and characteristics.

| **Dataset Name** | **Sensor** | **Spatial Resolution** | **Temporal Resolution** | **Data Variables** | **Source** |
| --- | --- | --- | --- | --- | --- |
| VIIRS Active fire | VIIRS (Suomi NPP, NOAA-20) | 375m | Daily | Fire Radiative Power (FRP), Brightness Temperature, Confidence Level, Acquired Date, Location | sftp://fuoco.geog.umd.edu |

Table A2. Hyper-parameters for different ML and DL models : *Optimal hyperparameters for each model across countries. (a) MLP: input size, number of nodes, number of layers, and batch size. (b) CNN: input size, number of nodes, number of layers, epochs, and batch size. (c) LSTM: input size, number of nodes, number of layers, epochs, batch size, and differencing. (d) CNNLSTM: step size, sequence size, number of nodes, kernel size, number of layers, epochs, and batch size. (e) ConvLSTM: step size, sequence size, number of nodes, kernel size, number of layers, epochs, and batch size.*

**a. MLP**

**b. CNN**

**c. LSTM**

**d. CNN-LSTM**

**e. CONV-LSTM**


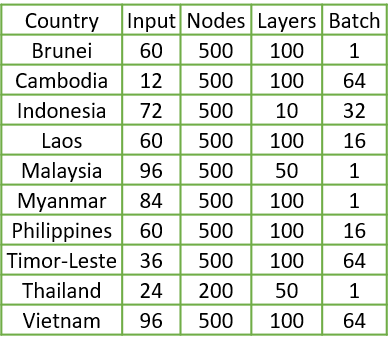

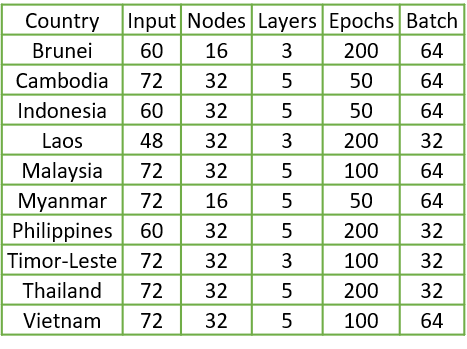

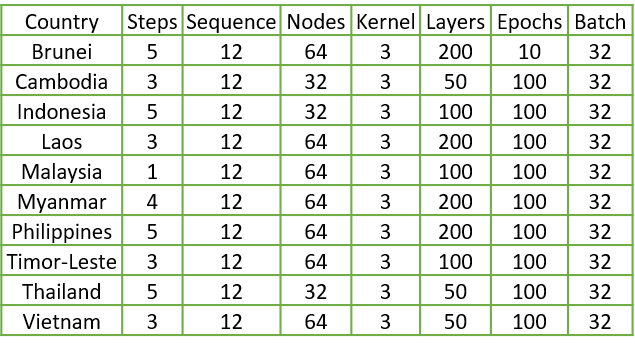


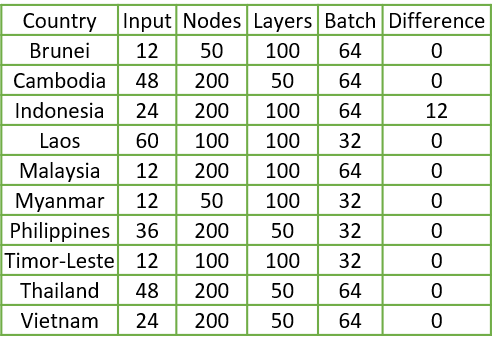


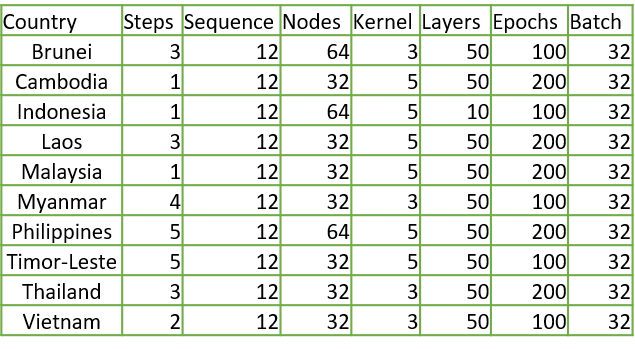


Figure S1. Fire prediction results for Indonesia and Malaysia.
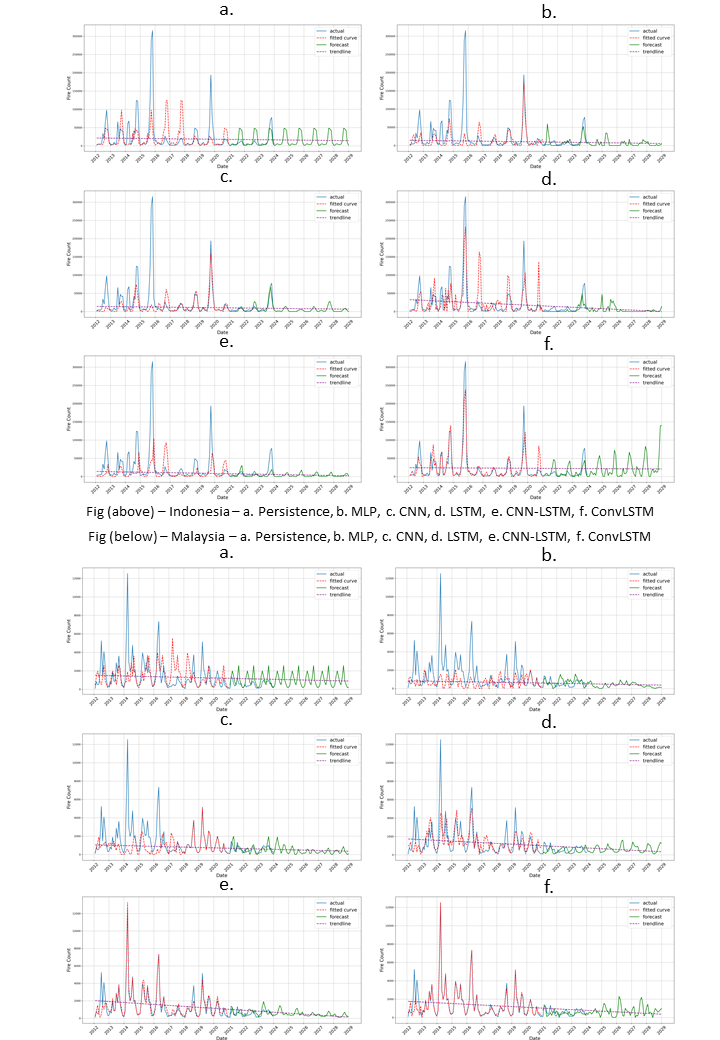


Figure S2. Fire prediction results for Myanmar and the Philippines


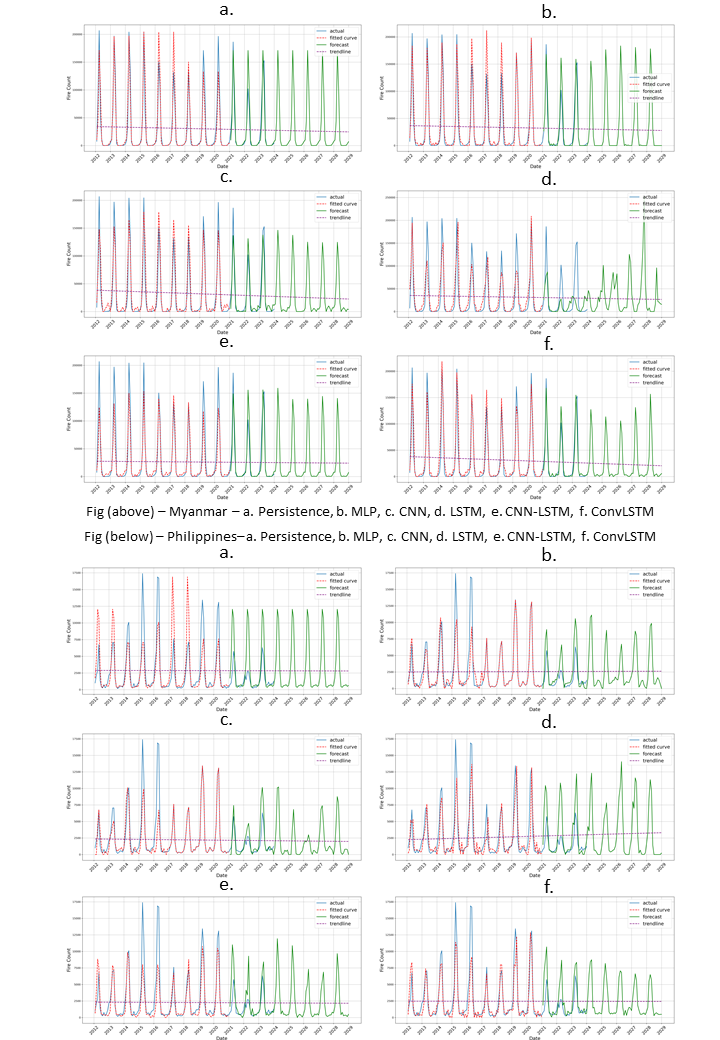


Figure S3. Fire prediction results for Timor Leste and the Vietnam
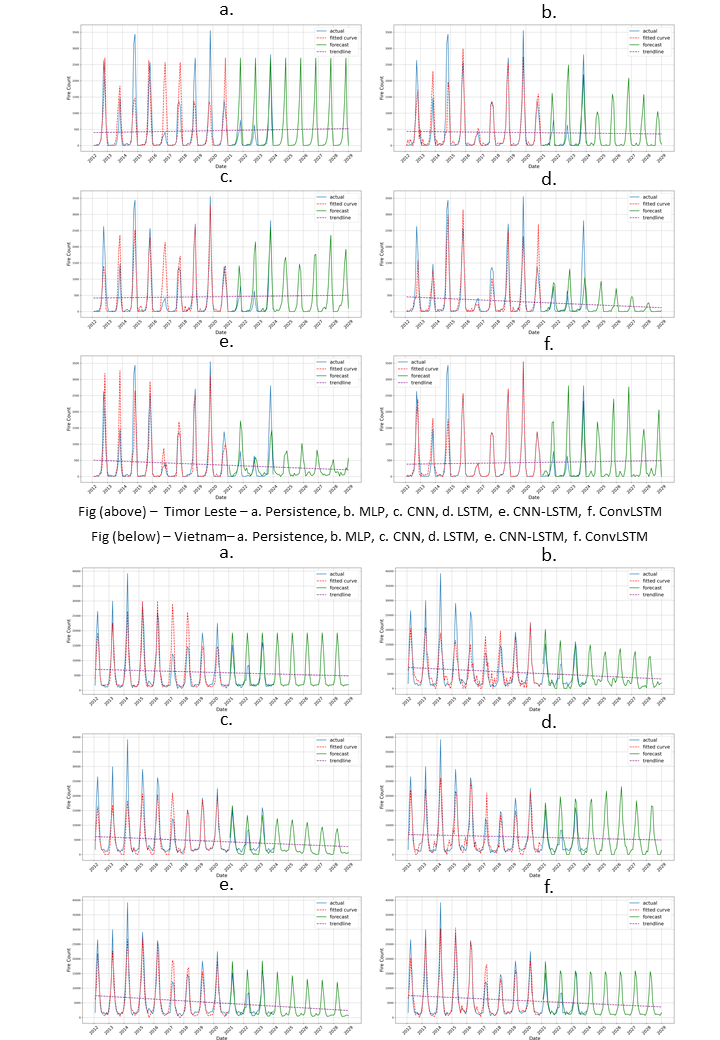

Supplement: Supplementary file 1 — Supplementary Information. [file 41598_2025_628_MOESM1_ESM.docx]
